# Serotonin suppresses cortical theta bursts during NREM sleep

**DOI:** 10.3389/fphar.2026.1767628

**Published:** 2026-03-25

**Authors:** Gergely F. Turi, Sasa Teng, Yueqing Peng

**Affiliations:** 1 Program in Memory Longevity, UT Southwestern Medical Center, Dallas, TX, United States; 2 Department of Neurology, Department of Pathology and Cell Biology, Vagelos College of Physicians and Surgeons, Columbia University, New York, NY, United States

**Keywords:** 5HT1A, dentate gyrus, dorsal raphe, EEG, NREM sleep, serotonin, theta burst

## Abstract

The monoaminergic neurotransmitter serotonin (5-HT) is one of key regulators of brain states, with reduced release during sleep. Serotonin is also known to powerfully suppress hippocampal theta oscillations in the awake brain. During non-rapid eye movement (NREM) sleep, brief theta bursts (TBs) have been identified in the cortex and are implied in neuronal reactivation. We investigated whether and how the 5-HT system modulates these cortical TB events during NREM sleep. Using simultaneous EEG/EMG recordings and fiber photometry to monitor 5-HT neuronal activity and extracellular 5-HT concentration, we found that cortical TB occurrence is inversely correlated with the serotonergic tone: calcium activity in serotonergic neurons and extracellular 5-HT levels gradually decreased before TBs, suggesting that TBs are most probable when 5-HT activity is low. Then, we demonstrated that optogenetic activation of dorsal raphe 5-HT neurons during NREM sleep significantly decreased the rate of TBs and reduced theta power in the absence of sleep-to-wake transitions. Conversely, pharmacological inhibition of the 5-HT system using an inhibitory 5HT1A receptor agonist increased the rate of TBs during NREM sleep. Furthermore, we examined TB-related hippocampal activity and found that the major excitatory neuronal populations in the dentate gyrus (DG), including granule cells and mossy cells, significantly increased calcium activity before the TBs. This TB-related DG activity increase was suppressed when the 5-HT system was pharmacologically inhibited. These results indicate that serotonin suppresses cortical theta bursts and that a low 5-HT state is essential for TB-related hippocampal activity during NREM sleep, defining a critical neuromodulatory gating mechanism for sleep-specific brain oscillation associated with memory processing.

## Introduction

Monoaminergic neurotransmitter systems are located throughout the hypothalamus and brainstem and are known to be involved in sleep and memory regulation. Among these systems, serotonin (5-HT) is originally considered to promote wakefulness (Jouvet, 1999) and known as a waking substance. It has been shown very early on that lesioning the raphe nuclei, where the 5-HT synthesizing neurons are located, leads to long lasting insomnia and the decrease of slow-wave sleep in cats (Jouvet, 1969). Subsequent electrophysiological studies revealed that stereotypical 5-HT neurons discharge tonically and regularly at high rates during waking, and at progressively slower rates during slow-wave sleep and virtually stop firing during paradoxical or REM sleep intervals (Sakai and Crochet, 2001). Interestingly, a recent study shows that optogenetic stimulation of dorsal raphe (DRN) neurons has bidirectional mode-dependent effects on sleep in mice: burst stimulation causes immediate wake while tonic stimulation slowly promote sleep (Oikonomou et al., 2019). More recently, imaging studies further confirmed that the serotonin level is the highest during awake periods, and slowly fluctuates following an infraslow cycle during NREM sleep (Kato et al., 2022; Turi et al., 2025). Together, these studies suggest the dynamic role of 5-HT system in regulating sleep and wakefulness.

Serotonin has more than a dozen of different receptor subtypes that mediate inhibitory or excitatory effects on pre and postsynatpic sites. Manipulation of 5-HT levels, or its receptors lead to altered sleep structure. For instance, increasing 5-HT level in the brain by the pharmacological blockade of 5-HT transporters inhibits REM sleep in rodents (Monaca et al., 2003) and humans (Hendrickse et al., 1994). Global removal of the inhibitory 5HT1A leads to altered sleep cycle by increasing REM sleep (Boutrel et al., 2002; Monaca et al., 2003; Gordon et al., 2005). In addition to their role in sleep regulation, serotonin receptors are broadly expressed across hippocampal subfields and along the dorsal-ventral axis, providing a substrate for complex modulation of excitatory and inhibitory transmission. Consistent with this, serotonergic signaling has been implicated in hippocampus-dependent functions including spatial memory formation and reward-related behaviors (Teixeira et al., 2018; Yoshida et al., 2019; Luchetti et al., 2020).

Serotonin is capable of modulating faster oscillations as well. Theta oscillations are the hallmark of awake brain activity, displayed robustly during locomotion and exploration in the hippocampus (Vanderwolf, 1969). Serotonin has a profound impact on theta as hippocampal theta oscillations can be suppressed pharmacologically by 5HT1A agonists (McNaughton and Coop, 1991) or genetic downregulation of these receptors. 5HT1A receptor knock out mice have an increased magnitude of theta oscillation during awake exploration and decreased ripple oscillation amplitude during slow-wave sleep epochs (Gordon et al., 2005).

Theta activity is also present during specific sleep stages in both humans and mice most prominently during REM stage. A less well-known form of theta activity has recently been described in the hippocampus and cortex. These events are manifested during NREM sleep as brief, 300–600 ms theta burst (TB) epochs both in human and rodent EEG recordings. In humans, TBs precede cortical downstates and more pronounced in NREM stage 2 (Gonzalez et al., 2018; Jiang et al., 2019). In mice, TBs have been shown to originate from midline thalamic areas and strongly linked to neuronal reactivation during NREM sleep (Xiao et al., 2024).

In this study, we combined EEG/EMG recordings with fiber photometry, pharmacology, and optogenetics, to investigate how the 5-HT system regulates cortical TB activity and TB-related hippocampal activity during NREM sleep. We found an inverse correlation between 5-HT activity and TBs: TBs were most frequent when the overall 5-HT neuronal activity or 5-HT level was low. Furthermore, this low 5-HT enables TB-related hippocampal activity during NREM sleep.

## Materials and methods

### Animals

All procedures were carried out in accordance with the US National Institute of Health (NIH) guidelines for the care and use of laboratory animals, and approved by the Animal Care and Use Committees of Columbia University. Male and female Slc6a4-Cre (also known as SERT-Cre, JAX #014554, 12–20 weeks old), male Dock10-Cre (MGI:6117432), male Drd2-Cre (MMRRC:032108-UCD), male C57BL/6 J mice (JAX #000664, 12–16 weeks old) were used for all experiments. Mice were housed in 12-h light-dark cycles (lights on at 07:00 a.m. and off at 07:00 p.m., temperatures of 65 °F–75 °F with 40%–60% humidity) with free access to food and water.

### Viral constructs

AAV1-Syn-Flex-GCaMP6s (#100845) and AAV1-EF1α-double-floxed-hChR2(H134R)-EYFP-WPRE-HGHpA (AAV1-DIO-ChR2, #20298) were obtained from Addgene. AAV9-hSyn-GRAB_5-HT2h_ was obtained from Vigene Biosciences (Cat# YL10097-AV9).

### Surgical procedures

#### EEG and fiber implants

Mice were anaesthetized with a mixture of ketamine and Xylazine (100 mg/kg and 10 mg/kg, *i. p.*), then placed on a stereotaxic frame with a closed-loop heating system to maintain body temperature. After asepsis, the skin was incised to expose the skull and a small craniotomy (∼0.5 mm in diameter) was made on the skull above the regions of interest. For EEG and EMG recordings, a reference screw was inserted into the skull on top of the cerebellum. EEG recordings were made from two screws on top of the cortex 1 mm from midline, 1.5 mm anterior to the bregma and 1.5 mm posterior to the bregma, respectively. Two EMG electrodes were bilaterally inserted into the neck musculature. EEG screws and EMG electrodes were connected to a PCB board which was soldered with a 5-position pin connector. For optogenetic stimulation or fiber photometry recording, an optical fiber (0.2 mm diameter, 0.39 NA, Thorlabs) were implanted into the dorsal raphe with the tip 0.1 mm above the virus injection site. All the implants were secured onto the skull with dental cement (Lang Dental Manufacturing). After surgery, the animals were returned to their home cages for recovery for at least 2 weeks before any experiment.

#### Virus injection

The viral prep solution containing 150–200 nL viral construct was loaded into a pulled glass capillary and pressure injected into the target region using a Nanoinjector (WPI). For fiber photometry, AAV expressing GRAB_5-HT_ or GCaMP6s were unilaterally injected in the dorsal raphe nucleus (AP -4.5 mm, ML 0 mm, DV 3.2 mm) or hippocampus (AP -1.9 mm, ML 1.5 mm, DV 1.7 mm). For optogenetic experiments, 200 nL AAV1 expressing channelrhodopsin was unilaterally injected into the dorsal raphe nucleus in Slc6a4-Cre mice. The dorso-ventral coordinates listed above are relative to the pial surface.

#### Sleep recording

Mouse sleep behavior was monitored using EEG and EMG recording along with an infrared video camera at 30 frames per second. Recordings were performed across 24 h (light on at 7:00 a.m. and off at 7:00 p.m.) in a behavioral chamber inside a sound attenuating cubicle (Med Associated Inc.). Animals were habituated in the chamber for at least 4 h before recording. EEG and EMG signals were recorded, bandpass filtered at 0.5–500 Hz, and digitized at 1,017 Hz with 32-channel amplifiers (TDT, PZ5 and RZ5D). Spectral analysis was carried out using fast Fourier transform (FFT) over a 5 s sliding window, sequentially shifted by 2 s increments (bins). Brain states were semi-automatically classified into wake, NREM sleep, and REM sleep states using a custom-written MATLAB (MathWorks) program as previously described (Teng et al., 2022; Turi et al., 2025). Accordingly, the scored states were the followings: wake: desynchronized EEG and high EMG activity; NREM: synchronized EEG with high-amplitude, delta frequency (0.5–4 Hz) activity and low EMG activity; REM: high power at theta frequencies (6–9 Hz) and low EMG activity. Semi-auto classification was validated manually by trained experimenters.

#### Theta burst detection

TB detection was previously described (Xiao et al., 2024). Specifically, the spectral power was re-analyzed using fast Fourier transform (FFT) over a 0.3 s sliding window, sequentially shifted by 0.125 s increments (bins). Relative theta power (4–10 Hz) was calculated by dividing the theta power in each time bin by the total EEG power averaged across the recording session. Then, we categorized theta burst (TB) events in the following way: the baseline of the theta power was calculated by averaging the signal, then a threshold of 3 standard deviation above the baseline was used to detect the TB events during NREM sleep. To remove false positive events caused by EEG noise, we applied a secondary criterion: the ratio of theta power over the whole power in each time bin should be larger than 1 standard deviation above the average. The TB rate refers to the number of TB events per 1-min NREM sleep. The onsets of the TBs, defined as the earliest time that the theta signal crosses the threshold, were used to align the photometry signals.

#### Fiber photometry

Fiber photometry recordings were performed as previously described (Turi et al., 2025). In brief, the GCaMP6s or GRAB sensor fluorescence was excited by sinusoidal modulated LED light (473 nm, 220 Hz; 405 nm, 350 Hz, Doric lenses) and detected by a femtowatt silicon photoreceiver (New Port, 2151). Photometric signals and EEG/EMG signals were simultaneously acquired by a real-time processor (RZ5D, TDT) and synchronized with behavioral video recording. A motorized commutator (ACO32, TDT) was used to route electric wires and optical fiber. The collected data were analyzed by custom MATLAB scripts. The photometry signals were first extracted then subjected to low-pass filter (2 Hz). A least-squares linear fit was then applied to produce a fitted 405 nm signal. The DF/F was calculated as: (F-F0)/F0, where F0 was the fitted 405 nm signal. To compare activity across animals, photometric data were further normalized using Z-score calculation in each mouse. To examine the activity before and after theta burst, the photometric signals were aligned to the onset of theta burst to generate the per-stimulus time histogram (PSTH).

#### Optogenetic manipulation

All optogenetic stimulation was conducted unilaterally. Mice were habituated in the behavioral chamber for at least 4 h before the experiment. Light pulses (1 Hz, 10 ms) with a duration of 10 s from a 473 nm laser diode (Shanghai laser & Optics Century Co., Ltd.) were controlled by a microcontroller board (Arduino Mega 2560, Arduino). Inter-stimulation interval for optogenetic stimulation was either 10 or 5 min. Laser power was set to 6–8 mW measured at the fiber tip. Optogenetic stimulation was evenly applied during both light and dark cycles while conducting fiber photometry recordings (1-h session with an interval of 1–3 h).

#### Pharmacology

5HT1A agonist 8-Hydroxy-DPAT hydrobromide (Cat#0529) was obtained from Tocris Bioscience. On the day of the experiment, mice were habituated and recorded in a behavioral chamber for 1-2 sessions (pre-recording). Following the pre-recording, 8-OH-DPAT (1 mg/kg in 0.9% NaCl, 200 µL) was administered intraperitoneally in the light cycles. Immediately after the injection, mice were recorded for another 2-h session (post).

#### Histology

Viral expression and placement of optical implants were verified after the termination of the experiments using DAPI counterstaining of 100 μm coronal sections (Prolong Gold Antifade Mountant with DAPI, Invitrogen). Images were acquired using a Zeiss 810 confocal microscope.

#### Statistics

Sample sizes were guided by prior experience with these assays and practical considerations rather than an *a priori* power calculation. Investigators were aware of group assignments, and animals were assigned to experimental groups without a formal randomization procedure. Animals were excluded only when *post hoc* histology indicated off-target viral expression or incorrect fiber placement. Statistical comparisons were performed using two-sided paired and unpaired t-tests, as well as one-way ANOVA, as specified in the corresponding figure legends. All analyses were conducted in MATLAB. Data are presented as mean ± SEM.

## Results

### Serotonergic activity and 5-HT concentration are anticorrelated with TBs during NREM sleep

**FIGURE 1:**
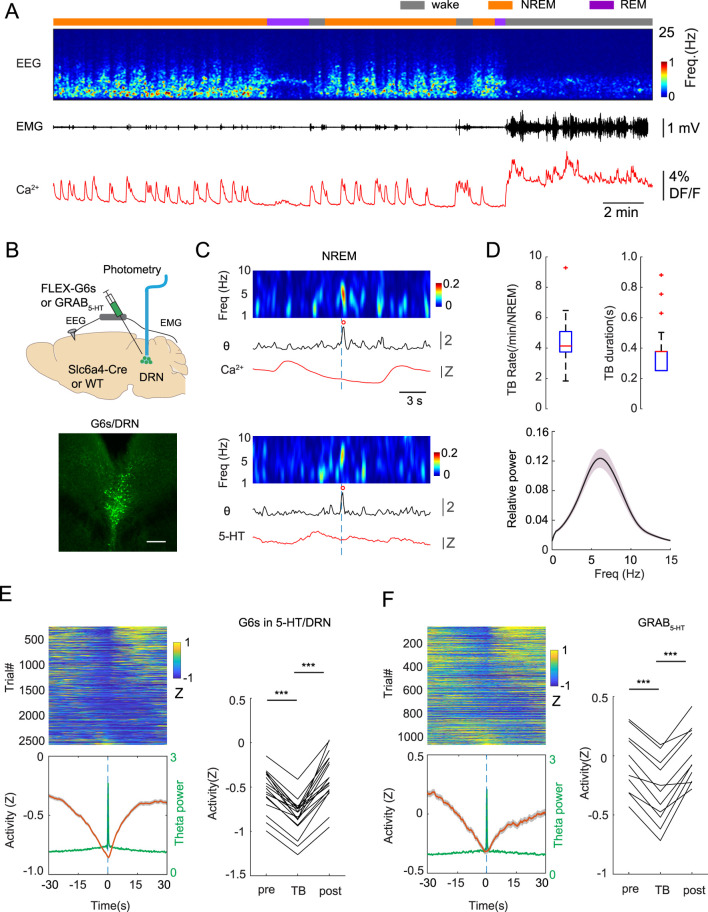
Theta burst (TB) during NREM sleep is anticorrelated with 5-HT activity. **(A)** A representative recording session showing 5-HT activity during wake and sleep cycles. From top to bottom, brain state, EEG spectrogram (0–25 Hz), EMG signal, photometry signal. **(B)** Top, Schematic of experimental design. Bottom, a fluorescent image showing GCaMP6 s expression in the DRN in a Slc6a4-Cre mouse. Scale bar: 100 µm. **(C)** Top, a representative example showing calcium activity of 5-HT neurons (red trace) during a TB event. Bottom, same as top but with the GRAB_5-HT_ sensor. Heatmap on the top represents EEG power (1–10 Hz), black trace (middle) represents relative theta power, red circle corresponds to TB. Dashed lines indicate TB onset. **(D)** Quantification of rate, duration and spectral power (bottom) of TBs during NREM sleep. **(E)** Top left: PSTH of sorted calcium activity of 5-HT neurons, bottom left: averaged 5-HT activity (red line) and averaged theta power (green line). Time 0 indicates the TB onset. Right: quantification of 5-HT activity (n = 4 mice, 18 sessions). **(F)** Left, Same as **(E)** but showing 5-HT level obtained from GRAB_5-HT_ sensor (n = 4 mice, 11 sessions). ***P < 0.001, paired t-test.

To explore the interplay between the serotonergic system and TBs during sleep, we injected Slc6a4-Cre mice with AAV1-Syn-Flex-GCaMP6s in the dorsal raphe nuclei (DRN) followed by fiber optic and EEG/EMG implantation. After 2 weeks of recovery, we performed simultaneous EEG/EMG and photometry recordings intermittently (1 h on, 1 h off) while mice experienced natural sleep and wake cycles in a behavioral chamber (Figures 1A,B). Spectral analysis of EEG signals revealed periodically increased power in the theta range during NREM sleep. The rate and length of these theta burst (TB) events were highly similar to those described previously^1^ (Figures 1C,D). Although activity of 5-HT neurons and 5-HT concentration are generally considered to be low during NREM sleep, recent work highlighted the presence of regularly occurring 5-HT fluctuations with a cycle in the infraslow regime (∼0.03 Hz) (Kato et al., 2022; Turi et al., 2025) that induces the level of 5-HT increase periodically. To assess whether TBs are modulated by the 5-HT fluctuation, we constructed peri-stimulus time histograms (PSTH) of the DRN serotonergic activity centered on the onset of TB events (Figure 1E). This analysis revealed that 5-HT activity gradually decreased before the TBs and then increased again after the TBs. Since calcium activity represents intracellular activity and may not fully align with 5-HT release, we repeated these experiments in wildtype mice injected with the GRAB_5-HT_ sensor in the DRN, which reports extracellular 5-HT levels. Results obtained with the 5-HT sensor confirmed our previous findings: extracellular 5-HT was the lowest before the onset of TBs (Figure 1F).

### Serotonin suppresses TBs during NREM sleep

**FIGURE 2:**
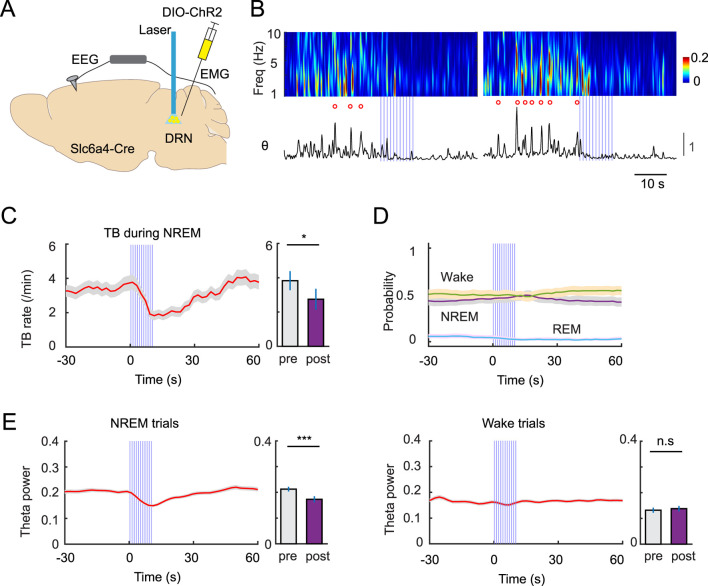
Optogenetic activation of raphe 5-HT neurons suppresses TBs during NREM sleep. **(A)** Schematic of experimental design. **(B)** Two representative examples showing EEG spectrogram (upper) and theta power (bottom) before and after optogenetic stimulation (1 Hz, 10 s). **(C)** Left, PSTH of average TB rate during NREM sleep before and after optogenetic stimulation. Right, quantification of average TB rate before (pre, time −10 s–0s) and after (post, time 10s–15 s) optogenetic stimulation (n = 38 sessions from 6 Slc6a4-Cre mice). Time 0 indicates the stimulation onset. **(D)** Probability of different brain states before and after optogenetic stimulation. Note the absence of major brain state changes. **(E)** Effect of optogenetic stimulation on theta power during NREM trials (left) and wake trials (right) (n = 38 sessions from 6 Slc6a4-Cre mice). n. s no significance, *P < 0.05, ***P < 0.001, paired t-test.

Based on the photometry experiments, we hypothesized that periodically increasing 5-HT tone during NREM sleep suppresses TB activity. To directly test this, we injected Slc6a4-Cre mice in the DRN with AAV1-DIO-ChR2 to express channelrhodopsin (ChR2) in 5-HT neurons (Figure 2A). DRN 5-HT neurons were then activated with a 1 Hz photosimulation light (duration of 10 s) during awake and sleep stages while EEG recording was performed (Figure 2B). The low stimulation frequency was selected to avoid possible effects on brain states when the 5-HT system is activated. We then analyzed the rate of TBs by aligning them with the onset of the laser stimulation. We found that optogenetic activation of 5-HT neurons during NREM led to a decrease in TB rate (Figure 2C) without significantly affecting overall brain states (Figure 2D). Consistently, we also observed decreased theta power during NREM sleep, following optogenetic activation (Figure 2E). Interestingly, optogenetic activation during wakefulness did not change theta power (Figure 2E).

### Pharmacological inhibition of the 5-HT system increases TB occurrence

**FIGURE 3:**
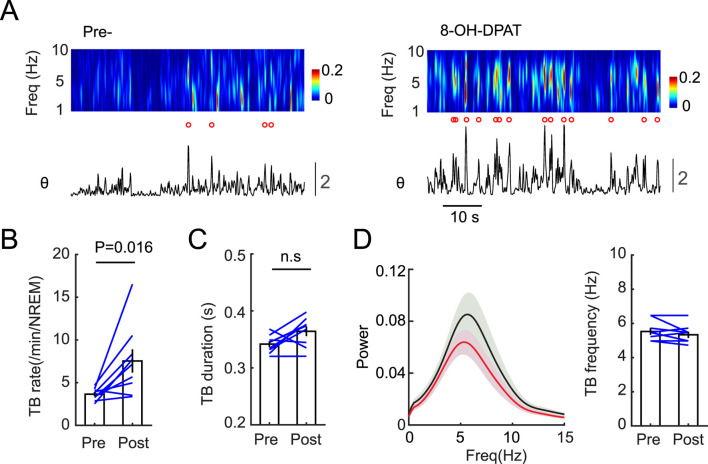
Pharmacological inhibition of 5-HT system enhances TBs during NREM sleep. **(A)** Representative examples showing EEG activity and theta burst occurrence before (Left, Pre-) and after the 5HT1A agonist (8-OH-DPAT) (1 mg/kg, Right). Red circles mark the above-threshold TB **(B)** Quantification of event rate shows significant increase in TB rate after 5HT1A agonist. **(C)** TB duration or **(D)** Spectral power of TB before (black) did not change significantly after (red) 5HT1A agonist. Right, quantification of peak frequency of TB before and after 5HT1A agonist. (N = 9 animals, paired t-test).

Next, we tested whether inhibiting the 5-HT system has effect on TBs. Considering the efficacy of viral expression used for optogenetic and/or chemogenetic manipulation, we decided to use systemic pharmacological inhibition in this study. Serotonin 1a receptor (5HT1A) is known as an inhibitory 5-HT receptor subtype within the 5-HT receptor family. It is also known to regulate the activity of 5-HT neurons as it is expressed by the 5-HT cells in the raphe as an inhibitory autoreceptor (Hjorth et al., 2000). We took advantage of this expression pattern and injected mice in the sleep recording chamber with the 5HT1A agonist 8-OH-DPAT (1 mg/kg, *i. p.*) to reversibly silence the 5-HT system. Analysis of EEG signals during NREM sleep after 8-OH-DPAT administration revealed increased rate of TBs (Figures 3A,B) while the duration and peak frequency of TBs did not change significantly (Figures 3C,D). These results suggest that inhibition of the 5-HT system is needed for TBs during NREM sleep.

### Modulation of serotonin on TB-related hippocampal activity during NREM sleep

**FIGURE 4:**
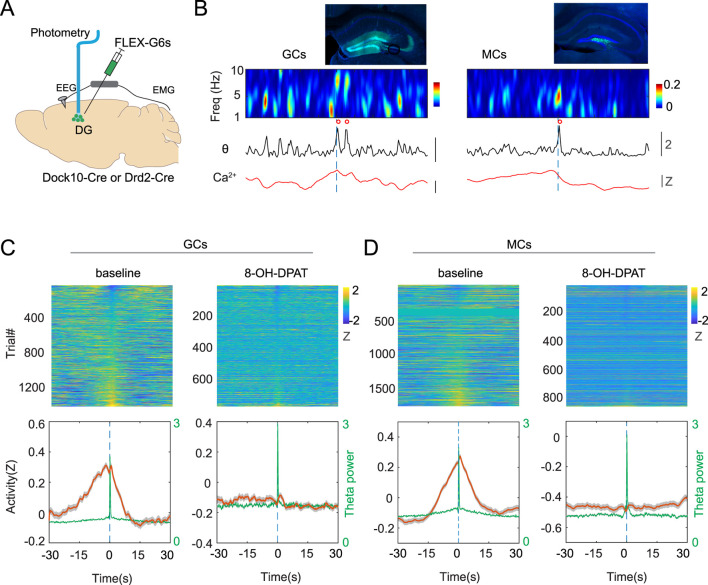
Serotonin modulates TB-related activity in the dentate gyrus. **(A)** Schematic of experimental design. **(B)** Two representative examples showing calcium activity in the granule cells (GCs, Left), and mossy cells (MCs, Right) in response to TB events (red circles). **(C)** Left, PSTH of baseline GC activity in response to TB during NREM sleep. Right, PSTH of GC activity after 5HT1A agonist during NREM sleep (12 sessions from 5 Dock10-Cre mice). Time 0 indicates the TB onset. Green traces represent the relative theta power. **(D)** PSTH of MC activity before (baseline) and after 5HT1A agonist (12 sessions from 4 Drd2-Cre mice).

The hippocampal formation, including the dentate gyrus, receives dense serotonergic innervation, and classical tracing studies together with more recent viral anatomical approaches indicate that this input arises predominantly from the raphe nuclei (Vertes et al., 1999; Muzerelle et al., 2016). Thus, serotonergic neurons are anatomically well positioned to influence hippocampal network dynamics during sleep. To investigate TB-related hippocampal activity, we recorded the activity of two major glutamatergic neuronal populations of the first input node of the hippocampal formation, the dentate gyrus (DG). We used Dock-10^Cre^ (Kohara et al., 2014) to genetically target DG granule cells (GCs), and Drd2^Cre^ mice (Senzai and Buzsaki, 2017) to target DG mossy cells (MCs). These transgenic lines were injected with AAV1-Syn-Flex-GCaMP6s and implanted with fiber optic, EEG and EMG probes (Figure 4A). Baseline recording of GCs and MCs revealed significant increase in calcium activity before the onset of TBs (Figures 4B–D). Strikingly, pharmacological inhibition of the 5-HT system suppressed TB-related calcium increase in both cell types (Figures 4C,D). These results imply that 5-HT modulation is needed for TB-related hippocampal activity during sleep.

## Discussion

The primary finding of this study is that serotonin suppresses cortical theta bursts (TBs) during non-rapid eye movement (NREM) sleep. This conclusion is supported by evidence showing an inverse correlation between the occurrence of cortical TBs and serotonergic tone: TBs were most frequent when the activity and extracellular concentration of 5-HT in the DRN were low. Establishing a causal link, optogenetic activation of DRN 5-HT neurons significantly decreased TB rate, whereas pharmacological inhibition of the 5-HT system using the inhibitory 5HT1A receptor agonist, 8-OH-DPAT, resulted in an increased frequency of TBs during NREM sleep. Furthermore, we demonstrated that a low 5-HT state is essential for TB-related hippocampal activity during NREM sleep, as the increase in activity of major excitatory neuronal populations in the DG observed during cortical TBs was suppressed when the 5-HT system was pharmacologically inhibited.

Long-term memory consolidation, known as systems consolidation, is largely supported by NREM or slow-wave sleep (SWS) (Lee and Dan, 2012; Brodt et al., 2023). This process requires the precisely synchronized occurrence of microarchitectural elements: neocortical slow oscillations (SOs), also characterized by alternating synchronized membrane hyperpolarization (downstates, DSs) and depolarization (upstates, USs), along with thalamocortical spindles and hippocampal sharp wave-ripples (SWRs) (Brodt et al., 2023). The triple coupling of these elements facilitates the distribution and transformation of newly encoded memories from the hippocampus to neocortical long-term storage sites. Cortical TBs are integral to this microstructure in humans, having been described as typically preceding cortical DSs and being more pronounced in NREM Stage 2 (Gonzalez et al., 2018). These cortical elements generally follow a sequential order of TBs → DSs → spindles → USs (Jiang et al., 2019). TBs, therefore, occur early in the cascade of events thought to facilitate cortico-hippocampal communication.

The TB suppression mechanism discovered here is directly linked to hippocampal function. We found that the activity of major excitatory neuronal populations in the DG, including GCs and MCs, significantly increased during cortical TBs. This TB-related increase in DG activity suggests that a low 5-HT state is a necessary condition for DG excitation during sleep. A plausible circuit mechanism for this effect is suggested by the serotonergic receptor landscape in the DG. GCs express both the inhibitory 5-HT1A receptor and the excitatory 5-HT4 receptor, whereas a subset of MCs expresses excitatory 5-HT2A receptors (Tanaka et al., 2012). Because MCs are among the first synaptic targets of GCs and in turn provide excitatory feedback to GCs as well as drive local inhibitory interneurons, serotonergic tone is well positioned to regulate the balance between recurrent excitation and feedback inhibition within the DG. In a relatively high-5-HT state, activation of inhibitory 5-HT1A signaling in GCs may dampen their recruitment, thereby limiting propagation through the GC–MC loop. By contrast, when serotonergic tone falls during NREM sleep, this inhibitory constraint may be relieved, allowing TB-related excitatory drive to recruit GCs more effectively and to engage downstream MC-mediated recurrent activity. Such a mechanism would be consistent with our observation that a low-5-HT state is permissive for TB-related DG activation, and it provides a cellular framework through which serotonergic fluctuations could gate hippocampal participation in this sleep microstate.

While the immediate focus of this study’s hippocampal component was the DG (which is upstream of CA3 and CA1), there is substantial literature connecting theta activity to memory reactivation events essential for consolidation. Specifically, brief, isolated theta waves occurring in the medial entorhinal cortex (MEC) during NREM sleep are consistently in concert with the reactivation of MEC cell ensembles (Xiao et al., 2024). These isolated theta waves often originate from the nucleus reuniens in the midline thalamus, which is involved in coordinating hippocampal-prefrontal interactions (Xiao et al., 2024).

Furthermore, reactivation of memory traces in hippocampal networks (involving CA1 and CA3 subregions) is classically coordinated by sharp wave-ripples (SWRs) (Klinzing et al., 2019). SWRs are temporally coupled to cortical events, typically coinciding with or occurring shortly after downstates (DSs). Since TBs precede DSs in the cortical sequence (Jiang et al., 2019), the suppression of cortical TBs by serotonin defines a critical neuromodulatory gating mechanism that determines when the cortex enters the synchronized state necessary for initiating the subsequent SWR-spindle-mediated consolidation dialogue.

One limitation of this study is the specificity of pharmacological inhibition with 5HT1A agonist, particularly its effect on DG activity. Due to the systemic treatment, it’s difficult to determine whether the suppression of TB-related DG activity is mediated by 5HT1A receptor in DG cells or elsewhere (e.g., raphe 5-HT neurons). Future studies with local pharmacological or genetic manipulation in the DG are needed to confirm the results.

In summary, this study defines a crucial neuromodulatory gating mechanism essential for organizing NREM sleep microstates associated with memory processing, demonstrating that serotonin suppresses cortical theta bursts (TBs). This serotonergic suppression is particularly significant considering the established role of NREM sleep in systems consolidation, which relies on precisely timed microarchitectural elements. By acting as a neuromodulatory brake on TBs, serotonin determines when the cortex shifts into the low-activity state necessary to initiate this subsequent consolidation sequence. The finding that a low 5-HT state is critical for DG activation during TBs mirrors existing data suggesting that serotonergic inputs, mediated by the inhibitory 5HT1A receptor, modulate rhythmic, infraslow oscillatory activity in the DG during NREM sleep and that inhibition of this system leads to contextual memory impairment (Turi et al., 2025). Our results, therefore, highlight how neuromodulators like serotonin finely control NREM microstates, gating specific network dynamics (TBs and associated DG activation) that precede the canonical SWR-spindle coordination underlying memory systems consolidation.

## Data Availability

The original contributions presented in the study are included in the article/Supplementary Material, further inquiries can be directed to the corresponding author.
